# Efficacy of Complementary Therapies in the Quality of Life of Breast Cancer Survivors

**DOI:** 10.3389/fonc.2017.00326

**Published:** 2018-01-11

**Authors:** Sahar Zaidi, Showket Hussain, Shalini Verma, Zubia Veqar, Asiya Khan, Sheeraz Un Nazir, Neha Singh, Jamal Ali Moiz, Pranay Tanwar, Anurag Srivastava, G. K. Rath, Ravi Mehrotra

**Affiliations:** ^1^Centre for Physiotherapy and Rehabilitation Sciences, Jamia Millia Islamia (Central University), New Delhi, India; ^2^National Institute of Cancer Prevention and Research, Indian Council of Medical Research (NICPR-ICMR), New Delhi, India; ^3^All India Institute of Medical Sciences (AIIMS), New Delhi, India; ^4^Institute of Clinical Sciences, Sahlgrenska University Hospital, Gothenberg University, Gothenberg, Sweden

**Keywords:** breast cancer complications, exercise, quality of life, aerobic training, resistance training

## Abstract

Breast cancer (BC) is the most common cancer diagnosed in women and the second most common cancer overall, ranking as the fifth cause of death from cancer. The chronicity of the disease produces long-term physiological and psychological manifestations, which adversely affect the quality of life of the individual. The primary treatment while managing cancer presents with various debilitating side effects. With the recent advances in treatment techniques that have improved the survival rate, patients suffer from continuing posttreatment complications. Patients seem to cope well with the stress of treatment of BC and sustain a normal life; however, the deterioration in physical well-being makes the patient functionally inefficient. Exercise has been proven to be an effective, safe, and feasible tool in combating the adverse effects of treatment, prevents complications and decreases the risk of BC-specific mortality. This review briefly presents an overview of the burden of the disease and its management strategies. Owing to the heterogeneity of the population and the multitude of therapies they receive, the response of each patient to treatment is different and so is the magnitude of adverse effects. The review discusses the late sequelae following treatment and evidence supporting the role of physical activity in their management. In conclusion, there is a need for personalized physical activity plans to be developed to suit the individual and their circumstances.

## Introduction

Breast cancer (BC) is the second most common cancer overall with 1.7 million new cases reported worldwide. With 883,000 cases occurring in less developed and 794,000 cases in more developed regions, it is the most common cancer diagnosed in women and ranks as the fifth cause of death from cancer overall (522,000 deaths) (1). The peak age of onset is between 40 and 50 years in Asian countries compared with the West (60–70 years) (2). Family history, female sex, age, and changing reproductive trends, including first childbirth after age of 30 years, early age at menarche, later menopause, and nulliparity, are its major independent risk factors (3). BC is classified based on the presence of three receptors found on cancer cells: the estrogen receptor (ER), progesterone receptor (PR), and human epidermal growth factor 2-neu (HER2) receptor. Hormone receptor (HR) positive BCs include expression of ER and PR, accounting for approximately 60% of all BC cases (4). The oncogene HER2 is overexpressed in around 20% of all cases while the remaining 20% are negative for the expression of ER, PR, and HER2, also known as triple-negative breast cancer (TNBC) (5, 6).

The primary treatment of BC includes surgery, adjuvant cytotoxic chemotherapy, radiotherapy, adjuvant endocrine therapy, neoadjuvant anti-HER2 therapies, and personalized medicine. Principle factors in establishing treatment procedure include patient’s age, menopausal status, comorbidities, histologic grade, lymphovascular spread, HR status, and HER2 overexpression (7). Patients with HR positive tumors typically receive endocrine therapy [e.g., selective estrogen-receptor response modulators and aromatase inhibitors (AI)] as one of the treatment options, however, when the disease becomes metastatic, all patients eventually develop endocrine resistance and require cytotoxic chemotherapy (8, 9). Significant advances in the treatment of patients with HER2 overexpressing tumors include targeted therapies that have improved the clinical outcomes for patients with metastatic disease and enhanced survival (10). Patients with TNBCs tend to display an aggressive phenotype, currently do not have targeted therapy options as a standard of care, and have only a limited amount of cytotoxic agents available to treat their disease (11). In addition, according to the International Expert Consensus on the Primary Treatment of Early Breast Cancer, radiotherapy was indicated in patients with four or more positive nodes and should be avoided in elderly and those with substantial comorbidity following breast conserving surgery (7).

Depending on the primary treatment conferred, cancer management might present with various debilitating side effects in 72–96% of cancer patients (12). Treatment-specific changes, along with the morbidity associated with the disease, can lead to impairments in physiological as well as psychological and behavioral attributes, eventually leading to limitations in the ability to execute daily activities and participate in social events (13). BC survivors experience treatment-related distress, fear of recurrence, changes in body image and sexuality, as well as physical toxicities that result from adjuvant therapies (14). Post treatment symptoms such as pain and fatigue often persist, and interfere with functional capacity (15). This impairment is reflected in the fact that BC is responsible for the highest number of disability-adjusted life years in women (13.1 million) (16), imposing an economic burden of over $88 billion (17). Such functional limitation directly affects quality of life (QOL) and should not be left untreated (15). Exercise has been proven to be an effective, safe, and feasible tool to combat these adverse effects of treatment. It has further been shown to prevent complications in BC patients (18, 19), including the risk of postmenopausal BC which is decreased by 12–29% (20–23), and that of BC-specific mortality which is reduced by 15–67% (24). In addition, exercise-induced adaptations and better muscular performance may attenuate cancer toxicities, which in turn could augment the cure rate, improve the QOL for cancer survivors, and may even increase long-term survival (25–27). However, owing to the heterogeneity of this population and the multitude of therapies they receive, the response of each patient to treatment is different and so is the magnitude of adverse effects. Therefore, the purpose of the present review is to discuss the evidence regarding the late sequelae following primary treatment and the role of physical activity in their management. It includes recommendations for future physical activity interventions in brief.

## Pain and Lymphedema

About 12–51% of patients complain of pain after treatment (28), which might be of the following two types: (i) musculoskeletal pain resulting from injuries to muscle and ligaments that usually heal and are more likely to be transient and (ii) neuropathic pain from damage to the nerve tissue, which may become a more persistent problem (29). The symptoms of pain tend to diminish with time, affecting 47% of patients 1–3 years following treatment and persisting in up to 30% of patients even after 5 years. In addition, pain in the arm and shoulder ranged between 9 and 68% and in the breast area between 15 and 72% post-surgery (30). Although the occurrence and severity are wide-ranging, the most significant predictor for pain was found to be age. Women aged less than 40 are 3.6 times more likely to report pain than women aged 60–69 years. Treatment was another key determinant with axillary dissection and radiotherapy resulting in significantly more pain, whereas the surgical procedure (breast conserving surgery vs. mastectomy) or use of chemotherapy showed no differences in pain outcomes (31).

Another sequel of BC treatment is lymphedema, affecting 6–43% of BC patients (32). It results from insufficient lymph transport caused by damage to the lymphatic vasculature by lymph node dissection and radiotherapy (33). The most significant risk factors are mastectomy, radiotherapy, axillary dissection, tumor positive lymph nodes (34), and young age (35). Lymphedema has been shown to cause considerable functional disability as a result of pain, swelling, heaviness, paresthesia, and overall reduced mobility of the affected limb (36–38). It has also been associated with physiological and psychological side effects, such as compromised immune function (39), anxiety, distress, and social inhibition (40).

### Role of Physical Activity

The mainstay of treatment for cancer-related lymphedema includes complex decongestive therapy, exercise, and skin care (41). Ahmed et al. (42) proposed physiological changes in lymphatic function because of exercise, which include stimulation of lymph flow from skeletal muscle pumping and cardiopulmonary system (43). In addition, exercise has been shown to improve venous hemodynamics of the upper extremities causing a reduction in swelling (44). The American College of Sports Medicine (ACSM) in agreement with previous studies (45–47) now accepts resistance training as a safe and effective intervention (48) to reduce lymphedema in BC patients (49, 50). Schmidt et al. (47) concurred that resistance training decreased the incidence and intensity of arm and hand symptoms, and lymphedema exacerbations, and improved muscular strength.

## Muscle Strength

An estimated 10–70% of BC patients reported considerable restrictions in arm and shoulder mobility, and 17–33% reported decreased muscle strength following primary treatment (including surgery, chemotherapy, radiotherapy, and endocrine therapy) (32). The wide variation in prevalence could be attributed to the differences in assessment methods (measured or self-reported), time since treatment and type of surgery, with mastectomy and radiotherapy showing greater impairments (30). Hand grip and lower extremity strength have been an established prognostic variable for disability and mortality in elderly populations (51, 52). In BC survivors, the association between handgrip strength and QOL has been reported (53). More than 10% deficit in handgrip strength, specifically, on the surgical side was observed in 40% of BC survivors post-mastectomy (54). A significant reduction in the tip to tip pinch, key pinch, and palmar pinch has also been observed before and after radiotherapy following modified radical mastectomy.

Reduced muscle strength has been attributed to the loss of lean mass, which (55) is associated with disability, deteriorated QOL, altered functional status, and increase in fatigue and falls (56, 57). Lower extremity strength is a significant predictor of cancer-related fatigue, and its reduction increases fracture risk in BC survivors (58, 59). Chemotherapy is correlated with the loss of lean mass, especially in the lower limbs (60), leading to impaired isometric and isokinetic strength capacity, as well as muscular fatigue (61) that deteriorates with time since the completion of treatment (62).

### Role of Physical Activity

Exercise interventions have demonstrated significant improvements in lean body mass (LBM) and muscular strength (63). Specifically, resistance exercises have been shown to be effective for increasing lower-limb muscular strength and preventing the loss of LBM. These effects can be explained by increased motor unit recruitment and firing rate (64, 65), causing neural adaptations that result in increased force development (66). A recent review (67) also found improvements in upper and lower-limb muscular strength after resistance exercise program.

## Bone Health

**Figure 1:**
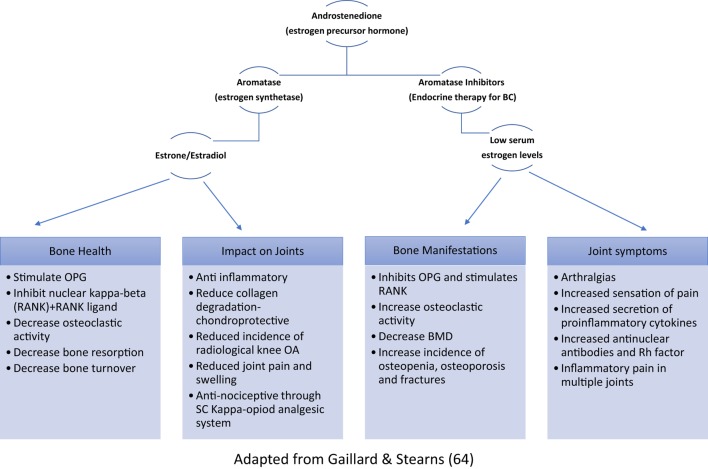
Role of estrogen in bone homeostasis and musculoskeletal symptoms (OPG, osteoprotegerin; RANK, receptor activator of nuclear kappa-beta; OA, osteoarthritis; SC, spinal cord; BMD, bone mineral density).

The rates of osteoporosis and osteopenia in cancer patients in remission were found to be 16 and 44%, respectively (68). Bone mineral homeostasis resulting from a balance between osteoblastic and osteoclastic activities has been found to be regulated by estrogen (69). Chemotherapy can inhibit bone proliferation directly and, along with ovarian suppression, indirectly reduce bone turnover *via* reduced estrogen (70) (Figure 1). Premature menopause is associated with an 11% reduction in bone mineral density (BMD), whereas chemotherapy and AI have shown to reduce BMD by around 4% in the lumbar spine (71–73). Therefore, these patients with increased risk of accelerated bone loss should have a baseline assessment of BMD (DXA-scan) within 3 months of ovarian suppression therapy and AI therapy, and 12 months post-chemotherapy amenorrhea. Furthermore, dietary supplementation with calcium and vitamin D, treatment with bisphosphonates and lifestyle advice should be incorporated into their management strategies (74).

### Role of Physical Activity

The potential negative side effects to body composition and bone loss from AIs may be diminished or eliminated through implementing regular physical activity and exercise (75). Aerobic exercise has shown to maintain total body BMD, and resistance training plus impact training preserved spinal BMD, posttreatment (76, 77). Upper-body resistance exercise recovers spinal density *via* tension produced by muscle insertion (78), whereas impact exercises activate hip and femur bone recovery through weight bearing by lower limbs (79).

## Arthralgias and Associated Symptoms

**Figure 2:**
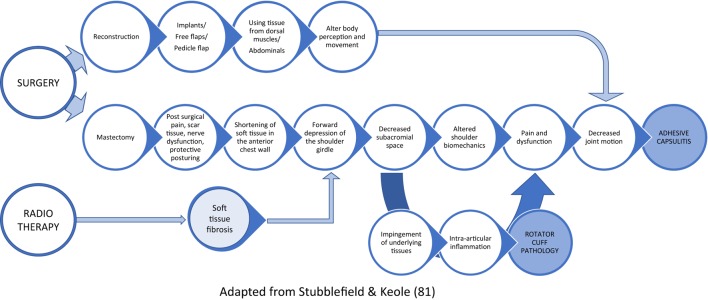
Shoulder dysfunction following breast cancer treatment.

Around 10–60% of BC patients report at least one upper-body symptom after surgery (80), and up to 61% report new or worsening joint symptoms following AI-treatment (81, 82), mediated by estrogen deficiency (Figure 1). These symptoms have been reported to significantly limit the performance of activities of daily living as well as work-related tasks (82, 83). AI-induced arthralgias were found to be severe enough to cause therapy interruption in up to 20% of patients (81, 84). The most commonly reported symptoms include morning stiffness and joint pain in the wrist (70%), hand (63%), knee (70%), back (54%), ankle/foot (51.8%), and hip (42.5%) (85). Restricted shoulder range of motion (ROM) was found in up to 50% of patients after treatment, with rotator cuff dysfunction and adhesive capsulitis being the commonest underlying pathologies (86). Surgery, specifically mastectomies or lymph node dissections, is an evidential risk factor for these complications and may cause axillary paresthesia, muscle dysfunction, and pain affecting the intercostal brachial or thoracodorsal nerve (Figure 2). Other frequently reported symptoms were digital stiffness, trigger finger, and carpal tunnel syndrome (81, 83, 87). AI when compared with tamoxifen increased patients’ predisposition to undergo surgery for carpal tunnel syndrome by up to seven times. Ultrasound and MRI evaluations have revealed fluid in the joint space and tendon sheath surrounding the digital flexor tendons and thickening of the tendon sheath (87, 88).

### Role of Physical Activity

Gentle articular movements and stretching, during and after treatments, promotes joint mobility and restores muscle flexibility (89, 90), prevents muscle contractures, and alters shoulder mechanics (91). Resistance exercise prevents musculoskeletal injury, improves muscular strength, improves ROM, and reduces body fat as well as systemic inflammation levels (75).

## Body Composition

Approximately 65% of all BC survivors are overweight or obese (92) with up to 84% reporting weight gain following diagnosis ranging from 2.5 to 5.2 kg (93). Sedentary lifestyle, postmenopausal status, intake of supportive medication, particularly glucocorticoids, slow metabolism, and endocrine manipulation predispose the individual to weight gain (94). Obesity and sedentary lifestyle are not only the causative factors for 25 and 33% of all BC cases (95) but also associated with poorer outcomes after a diagnosis, such as increased recurrence and total mortality. High level of fat mass decreases the survival rates of postmenopausal patients (19). Breasts are the primary site for estrogen production and produce pro-inflammatory cytokines and pro-tumorigenesis proteins, which are related to a poor quality of survival (18, 95). A 35% higher risk of BC-related death and a 41% higher risk of death due to other causes (96) have been noted in patients with obesity. In addition, BC treatment is related to increases in body fat as well as decreases in LBM and BMD (97, 98).

### Role of Physical Activity

A combination of aerobic and resistance training was most effective in reducing fat mass and raising LBM, as compared with aerobic exercise only (99, 100). In addition, performing resistance training twice a week for 6 months can increase LBM by 1–2 kg, a change that may prevent or reverse age-associated lean mass losses (101). It has been observed that changes in body composition and body weight take place only after 20 weeks of intervention (63). In postmenopausal women, exercise reduces body fat mass, which is associated with reduction in waist–hip ratio, serum estradiol, and inflammatory biomarkers levels (102, 103). Exercise and training increase muscle mass, which is correlated with a higher basal metabolism (104) promoting the transformation of white fat mass into brown fat mass (105).

## Physical Fitness

Breast cancer patients report reduced physical capacity (approximately 30%) (106, 107) compared with age- and sex-matched sedentary individuals. Peak oxygen uptake (VO_2_peak) reduces to 5–10% with ongoing chemotherapy (26, 108) and remains, on average, 22% lower in BC survivors despite normal cardiac function (as indicated by left ventricular ejection fraction ≥50%) (109). This implies that the decline in cardiorespiratory fitness could be attributed to other components of oxygen transport (i.e., pulmonary, hematologic, vascular, and skeletal muscle function) (109). This chemotherapy-induced reduction in VO_2_peak, spanning over a period of 12–24 weeks, is equivalent to that reported with 30 years of normal aging (109, 110). The evidence of a relationship between VO_2_peak and risk of cancer-related death in females and with BC-specific death also exists (111, 112).

**Figure 3:**
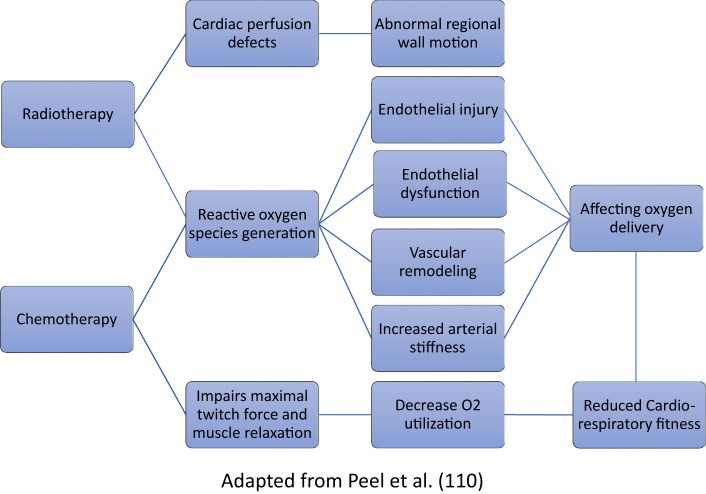
Proposed pathways for decline in cardiorespiratory fitness following breast cancer treatment.

Radiotherapy can impair the pulmonary gas exchange, as incidental radiation to the lungs can cause fibrosis (113). Both radiotherapy and chemotherapy for BC, especially anthracycline-containing chemotherapy, have shown to hamper both oxygen delivery and oxygen utilization (114). The proposed mechanisms have been outlined in Figure 3. Anemia may develop while undergoing therapy (115) and reduce oxygen delivery to muscle cells (116). It is evidential that exercise training is an effective intervention to improve cardiorespiratory function as well as QOL, strength, body composition and symptoms of fatigue, and depression in BC patients (63).

### Role of Physical Activity

Interventions that have improved VO_2_peak have typically lasted 8–24 weeks (117) and involve a prescription of 20–45 min of aerobic exercise two to three times per week at moderate intensity [65% maximum heart rate (MHR)] that may progress to vigorous intensity (>85% MHR) (117, 118). Many studies have also included resistance training. These varied prescriptions have resulted in improvements in VO_2_peak ranging from 2 to 32%, implying improved cardiorespiratory fitness of these patients (117, 118).

## Cardiovascular Dysfunction

Breast cancer patients are more prone to develop cardiovascular diseases, including heart failure and myocardial infarction compared with women who do not have a BC diagnosis (119, 120). Primary treatment may lead to cardiovascular impairments such as acute reductions in red blood cells and cardiotoxicity after treatment with anthracyclines (more permanent) or trastuzumab (reversible) (26, 108). A total of 13–39% of BC patients treated with anthracyclines present with cardiac events at some point in their life (106, 114, 121) (Figure 3). Radiotherapy at the left side increases cardiovascular mortality by 25% 15 years after diagnosis compared with women irradiated in the right side (122) and can also lead to valvular disease and accelerated coronary artery disease (123). Furthermore, radiotherapy-induced cardiotoxicity is amplified using adjuvant systemic chemotherapy, particularly anthracycline-based regimens (124) and newer agents such as trastuzumab (more commonly known as Herceptin) (125).

### Role of Physical Activity

Low-intensity aerobic exercise increases left ventricle volume, contractility, and elasticity of the cardiac muscle. Scott et al. inferred that exercise improves muscle cardiac irrigation (117) and reduces global low-grade inflammation, which is associated with cardiovascular diseases.

## Cardiovascular Risk Factors

Commonly encountered modifiable risk factors prevalent in BC patients include hypertension, dyslipidemia, overweight, and obesity and raised blood glucose or diabetes. Hypertension occurs in 25–50% of survivors (126) and is twice as common than age-matched female controls (127). Physical activity during and after BC treatment have consistently shown drops in systolic and diastolic blood pressure by up to 4.6–4.4 mmHg (128, 129). Aerobic training spanning 8–16 weeks with sessions lasting at least 20 min performed two to three times per week at moderate to vigorous intensity played an instrumental role in reducing blood pressure in BC survivors (63, 128).

On an average, BC women are likely to have high total cholesterol, triglycerides, and low-density lipoprotein (LDL) levels and lower high-density lipoprotein levels even before primary treatment, as compared to healthy, age-matched women (130, 131). Chemotherapy treatment may also increase triglyceride levels (132), however, tamoxifen decreases total cholesterol and LDL levels (133). Patients with type II diabetes mellitus are twice more susceptible to develop BC compared with age-matched women (127) and this increased risk has been proposed to be associated with weight gain (134). Moreover, studies on multiple GDM (gestational diabetes mellitus) pregnancies and BC risk (135, 136) suggest that abnormalities in glucose metabolism result in increased bioactivity of insulin-like growth factors influencing breast tissue remodeling and contributing to the initiation and progression of BC (137, 138). A combination of aerobic and resistance training has reported significant improvements in fasting insulin and blood glucose levels (139).

## Modes of Physical Activity

Previous studies examining the effect of physical activity are extremely varied in terms of (i) mode including aerobic, resistance, tai chi, and combined training (aerobic+resistance), (ii) frequency ranging from one to five times per week, (iii) length of a session between 15 and 90 min, and (iv) total duration of training spanning from 4 to 52 weeks.

### Aerobic Training

The meta-analysis by Schmid and Leitzmann (140) indicated that the moderate physical activity of 150 min/week after diagnosis is associated with a 24% reduction in total mortality among BC survivors and a 28% decrease in the risk of total mortality. Similar studies have also found statistically significant association between higher levels of physical activity and reduced risk of BC mortality (141, 142).

Aerobic exercise training improves metabolic function, functional capacity, and immune system, thereby diminishing the side effects before, during, or after cancer treatment (103, 143, 144). Despite these positive effects, a significant reduction in the QOL of BC survivors has been noted. Therefore, ways to make the aerobic exercise training effective for a long period of time are warranted. Cancer events such as diagnosis and completion of primary treatment have been proposed as unique windows of opportunity or “teachable moments” that can be used to influence behavior (145). Drum et al. (146) advised that post-cancer treatment aerobic training may be implemented based on the ACSM aerobic exercise guidelines for sedentary healthy persons. Exercise prescription must be based on cardiopulmonary exercise testing to make the training parameters more precise.

### Resistance Training

Most of the studies followed the ACSM recommendations focusing on large muscle groups (chest, back, shoulders, arms, buttock, hips, thighs, and calves) performing 1–3 sets of 8–10 repetitions, 2–3 days/week at an exercise intensity of 60–70% of one repetition maximum (RM) (48, 147). Owing to considerable variability in the implementation and responses to resistance training in different cancer populations and within patients of the same cancer type, a baseline assessment of strength is essential to individualize prescription. This mandates the highly individualized nature of exercise prescription in an oncology setting, and the need to investigate different doses, frequencies, duration, or load of resistance training (148).

The principle of specificity emphasizes that the training session is designed in view of the desired goals. The principle of overload mandates the exercise stimulus must be adequate to stress the system to a point where adaptations occur without disrupting the homeostasis and increasing the fatigue and risk of injury (149, 150). The incorporation of progression is critical because the body quickly adapts to a given exercise stress; the training stimulus must be gradually increased for continued development (151).

## Complementary Therapies and Alternative Medicine (CAM)

NIH has classified CAM into four categories: (1) mind and body medicine (meditation, yoga, acupuncture, guided imagery, qi gong), (2) manipulative and body-based practices (spinal manipulation, massage therapy, chiropractic medicine), (3) alternative systems (traditional Chinese medicine, Native American healing systems, Reiki, homeopathic medicine, Ayurveda), and (4) natural products (herbal supplements, botanical supplements, single supplements, and combinations of vitamins or minerals) (152). In addition to these, electrotherapeutic modalities, such as LASER, electrical stimulation, microwave diathermy, and thermotherapy have demonstrated insufficient evidence to support their use (153). Interventions with some evidence of efficacy in lymphedema include compression bandaging (154), pneumatic compression pumps (155), and decongestive therapies (154). Furthermore, the non-pharmacological management of pain with the use of transcutaneous electrical neuromuscular stimulation (TENS), acupuncture, or acupressure has been documented (155–157). However, there is a dearth of high-quality RCT’s to conclusively establish their effectiveness and dosimetry.

### Yoga

Yoga is a mind-body exercise program that provides physiological effects that are similar to aerobic exercise, including physical poses, breathing, and meditation (158, 159). It is widely accepted as an integrative form of therapy for BC (160). Its efficacy has been established both on and off treatment, and used as an adjunct to primary BC treatment (161). The main types of yoga used among cancer patients are Iyengar yoga, restorative yoga, and hatha yoga (162). 20–60 min of yoga per session for 4–24 weeks has been found to be an effective complementary modality of treatment in improving physical and psychosocial symptoms (160). Yoga practicing patients had better outcomes in terms of improved sleep quality, duration, latency, efficiency (163), improved overall QOL (164), reduced fatigue (165), menopausal symptoms (166), reduced body fat (167), and reduced depression scores (168).

It is usually the union of body and mind that is achieved in yoga (160). Mindfulness practices can address the cognitive and emotional components of pain and are associated with better coping skills, overall well-being, and spiritual development after chemotherapy, radiotherapy, and systemic treatment (164). Although there is no clear evidence how yoga induces relaxation, it has been postulated that yoga may lower stress induced arousal in BC patients in addition to increasing proprioception of somatic symptoms, building inner awareness, altering perceptions and mental responses to both internal and external stimuli (169). But there is a need to substantiate the genuinity, sustainability, and overall impact of yoga on BC survivors. A standardized intervention protocol which includes at least 18 h of yoga practice and is spread over at least 1 month, using a combination of selected asanas (physical postures adapted to the abilities of cancer patients), pranayamas (breathing exercises) and dhyanas (meditation) would be helpful for future recommendations (160). Yagli et al. found yoga with aerobic training to be more beneficial than aerobic exercise alone and recommended the incorporation of mind-body exercises in oncologic rehabilitation of patients who survive BC (164).

## Research Gaps and Future Perspectives

The major limitation of the present article was that it did not systematically review the literature, which makes the findings susceptible to selection bias. However, to control for these threats, the authors assessed the quality of selected studies using the PEDRO score which is a reliable and valid measure of methodological quality of RCTs (170, 171) and included findings from good-excellent quality RCTs, i.e., PEDRO score ≥ 5/11 (Table 1). Furthermore, for the most prevalent comorbidities, we have tried to summarize the efficacy of various exercise interventions (Table 2). However, a more quantitative efficacy analysis of each intervention may be separately performed comparing their effect size and 95% confidence interval, which was beyond the scope of the present study. A systematic review would provide more robust findings and an objective understanding of the efficacy of physical activity in combating treatment complications following BC. Future high-quality studies should also seek to explore training characteristics other than the standard ACSM exercise prescription, investigate the dose–response relationship between exercise and outcome variables, and finally draw comparisons with the traditional guidelines to design an optimal training protocol. While designing these programs, it is necessary to ensure optimal timing, and that all alternative exercise modes offer sufficient training stimulus in accordance with specific cancer site.

**Table 1:** Quality of studies assessing efficacy of exercise interventions.

Reference	Intervention groups	Outcomes	Quality of evidence^a^
Kilbreath et al. (89)	Stretching+PRE (*n* = 81) Control group (*n* = 97) 8 weeks	Sh. ROM ↑, Sh. strength ↑, lymphedema↔, QOL↔.	9/11

Lee et al. (91)	Scapula-oriented exercises (*n* = 16) General exercises (*n* = 16) Control group (*n* = 18) 8 weeks	Sh. strength↔, ROM ↑, Sh. disability↔, pain (VAS↔, BPI ↑), depression↔, QOL↑.	8/11

Fairey et al. (103)	RT (*n* = 25) Control group (*n* = 28) 15 weeks	CRP↔, RHR↔, HRR↑, SBP↔, DBP↔, HDL↔, LDL↔, TG↓, TC↔, TC: HDL↔	9/11

Schmitz et al. (104)	Immediate resistance (*n* = 42) Delayed resistance (*n* = 43) 12 months	BW↔, BMI↔, WC↔, FG↔, IGF II↓, insulin resistance↔, BF%↓, FFM↑, 1RM↑, insulin↔, LB 1RM↑.	8/11

Scott et al. (117)	AE+RT (*n* = 47) Control group (*n* = 43) 6 months	BW↓, BMI↓, WC↓, WHR↓, BF%↔, VO2Max↑, RHR↔, SBP↔, DBP↓, QOL↑, CRP↔, TC↓, estrone↔, estradiol↔, IGF↔, leptin↓, IGBP1, 3↔, testosterone↔, SHPG↔, insulin resistance↔.	8/11

Nuri et al. (139)	AE+RT (*n* = 14) Control group (*n* = 15) 15 weeks	FI↓, FG↓, insulin resistance↔, HDL↑, TG↓, VO2Peak↑, RHR↓, SBP↓, BW↓ BMI↓, WHR↓	6/11

Hughes et al. (167)	Yoga exercises (*n* = 31) Comprehensive exercises(*n* = 31) Control group (*n* = 32) 6 months	BW↔, BMI↔, RHR↔, SBP↔, DBP↔, VO2Max↔, arm strength↔, torso strength↔, arm volume, BF%↓, leg strength↑, flexibility↑, ROM↑.	5/11

Ahmed et al. (42)	RT (*n* = 42) Control group (*n* = 43) 6 months	Lymphedema↔, UL 1RM↑, LL 1RM↑	5/11

Vardar Yag˘lı et al. (164)	AE (*n* = 28) Y+AE (*n* = 32) 6 weeks	6 MWT↑(FC), strength↑, fatigue↓, QOL↑	6/11

Courneya et al. (26)	RT (*n* = 82) AE (*n* = 82) Control group (*n* = 78) 9–24 weeks	QOL↑ (R > A), fatigue↔, depression↔, VO2Max↑, (A > R), body fat% ↓(A > R), strength ↑(R > A), LBM ↑(R > A)	8/11

Friedenreich et al. (102)	AE (*n* = 160) Control group (*n* = 160) 12 weeks	Estrone↔, estradiol↓, androstenedione↔, testosterone ↔, SHBG ↑	8/11

Irwin et al. (75)	AE+RT (*n* = 61) Control group (*n* = 60) 12 months	Arthralgia↓, pain↓, disability↓, VO2Max↑, grip strength↑, BW↓, physical activity↑	7/11

Kim et al. (45)	PT+RT (*n* = 20) PT (*n* = 20) 8 weeks	Lymphedema ↓, QOL ↑	6/11

Nelson (101)	RT (*n* = 20) Control group (*n* = 19) 12 months	BMD↑, strength↑, muscle mass↑, balance↑	7/11

Winters-Stone et al. (77)	RT+Impact training (Power) (*n* = 52) Control group (*n* = 54) 12 months	Spine BMD ↑, osteocalcin↔, deoxypyrodinoline ↓	9/11

Winters-Stone et al. (78)	Power (*n* = 35) Control group (*n* = 36) 12 months	Hip and spine BMD ↔, Body fat% ↓, bone turnover ↔, upper-body strength ↑, LBM ↔, Strength↔	8/11

Sagen et al. (46)	RT (*n* = 104) Control group (*n* = 100) 6 months	Arm volume ↔, pain ↓	8/11

Schmitz et al. (47)	RT (*n* = 71) Control group (*n* = 70)	Bench press ↑, leg press ↑, BW↔, BMI↔, BF%↔, FM↔, LBM↔, lymphedema symptoms↓, lymphedema symptoms severity ↓	8/11

*PRE, progressive resistance exercise; QOL, quality of life; VAS, visual analog scale; BPI, Brief Pain Inventory; CRP, C-reactive protein; RHR, resting heart rate; HRR, heart rate recovery; SBP, systolic blood pressure; DBP, diastolic blood pressure; HDL, high-density lipids; LDL, low-density lipids; TG, triglycerides; TC, total cholesterol; BW, body weight; BMI, body mass index; WC, waist circumference; FM, fat mass; LBM, lean body mass; Sh., shoulder; RT, resistance training; AE, aerobic exercise; FG, fasting glucose; IGF II, insulin-like growth factor; BF%, body fat%; FFM, fat-free mass; RM, repetition maximum; LB, lower body; WHR, waist hip ratio; IGBP, immunoglobulin-binding protein; SHBG, sex hormone binding globulin; FI, fasting insulin; FG, fasting glucose; ROM, range of motion UL, upper limb; LL, lower limb; BMD, bone mineral density. ↑ sig. increase; ↓ sig. decrease; ↔ no sig. change*.

*^a^Methodological Quality of RCTs was assessed using the PEDRO score*.

**Table 2:** Strength of evidence of various exercise interventions for Breast cancer-related morbidities.

Outcome measure	Intervention	PEDRO score^a^	Strength of evidence
Pain and arthralgia	Scapular exercises	8	Good
	RT	8	Good
	AE+RT	7	Good
Lymphedema	RT	8	Good
	PT+RT	6	Good
Strength	Stretching+PRE	9	Excellent
	RT	5–8	Fair-Good
	POWER	8	Good
	AE+Y	6	Good
	AE+RT	7	Good
ROM	Stretching+PRE	9	Excellent
	Scapular	8	Good
	Y	5	Fair

**Body composition**			
BW	AE+RT	6–7	Good
BMI	AE+RT	6	Good
	POWER	8	Good
BF	AE+RT	6	Good
	Y	5	Fair
LBM	RT	7/8	Good
BMD	RT	7	Good
	POWER	9	Excellent
Lipid			
TC	AE+RT	6	Good
HDL	AE+RT	8	Good
TG	RT	9	Excellent
	AE+RT	6	Good

**Cardiovascular fitness**			
VO2 max	AE	8	Good
	AE+RT	6–8	Good
RHR	AE+RT	6/8	Good
DBP	AE+RT	8	Good
SBP	AE+RT	6	Good
FC	AE+Y	8	Good

**Hormones**			
Estradiol	AE	8	Good
SHBG	AE	8	Good
Leptin	AE+RT	8	Good
FBG	AE+RT	6	Good
FATIGUE	AE+Y	6	Good
QOL	Scapular Ex	8	Good
	AE+RT	8	Good
	AE+Y	6	Good
	RT	8	Good
	PT+RT	6	Good

*Y, yoga; PT, physiotherapy; FBG, fasting blood glucose; FC, functional capacity; QOL, quality of life; RHR, resting heart rate; SBP, systolic blood pressure; DBP, diastolic blood pressure; HDL, high-density lipids; TG, triglycerides; TC, total cholesterol; BW, body weight; BMI: body mass index; LBM: lean body mass; BMD: bone mineral density; RT: resistance training; AE, aerobic exercise; BF, body fat; SHBG, sex hormone binding globulin; ROM: range of motion*.

*^a^PEDRO score: scale for assessing methodological quality of RCTs (170, 171)*.

This review briefly outlines the ongoing primary treatment options available for BC population, prevalent musculoskeletal complications, and appropriate physical activity measures that can be adopted to combat the same, consequently, emphasizing the role of exercise post-primary treatment in BC survivors to improve overall QOL and reduce mortality. The intent is to encourage BC survivors to adopt a physically active lifestyle as part of the path to recovery. The survivors mainly face the challenge of initiating, reinitiating, and maintaining the activity levels, owing to confounding factors, such as personal, physiological, psychosocial, immunological, and endocrinological. To remedy this, tailor made exercise programs should be designed that embrace the interests, needs, capabilities, and preferences of patients. These personalized programs should offer a variety of alternative exercise modes in order to cater to specific outcome measures (strength, physical fitness, fatigue), help combat treatment-related adverse effects (decline in muscle mass, bone density, physical function, and psychological well-being), and maximize adherence to treatment, thus enhancing their efficacy.

To conclude, it is imperative to mention that the need of the hour is to explore the possibilities of above therapies to combat the deadly disease and most importantly the complications associated with cancer to destigmatize the social taboos associated with it. A detailed, focused and evidence-based research should be carried out to establish the efficacy of individual therapy to benefit the patient population. In addition to this, there should be awareness about Human papilloma virus vaccination, cancer websites, one to one interaction, government schemes, and government sponsored treatment modalities to improve its acceptability and affordability.

## Author Contributions

SZ and SV were involved in the primary writing of the manuscript. SH conceptualized the paper and guided the writing of the manuscript. SH also contributed to regular manuscript corrections and revisions of content. SN, NS, AK, AS, GR, ZV, JM, PT, and RM were involved in critical revision of the manuscript. All authors read and approved the final manuscript.

## Conflict of Interest Statement

The authors declare that the research was conducted in the absence of any commercial or financial relationships that could be construed as a potential conflict of interest.
